# Water Quality Conditions Associated with Cattle Grazing and Recreation on National Forest Lands

**DOI:** 10.1371/journal.pone.0068127

**Published:** 2013-06-27

**Authors:** Leslie M. Roche, Lea Kromschroeder, Edward R. Atwill, Randy A. Dahlgren, Kenneth W. Tate

**Affiliations:** 1 Department of Plant Sciences, University of California, Davis, California, United States of America; 2 School of Veterinary Medicine, University of California University of California, Davis, California, United States of America; 3 Department of Land, Air, and Water Resources, University of California, Davis, California, United States of America; U. S. Salinity Lab, United States of America

## Abstract

There is substantial concern that microbial and nutrient pollution by cattle on public lands degrades water quality, threatening human and ecological health. Given the importance of clean water on multiple-use landscapes, additional research is required to document and examine potential water quality issues across common resource use activities. During the 2011 grazing-recreation season, we conducted a cross sectional survey of water quality conditions associated with cattle grazing and/or recreation on 12 public lands grazing allotments in California. Our specific study objectives were to 1) quantify fecal indicator bacteria (FIB; fecal coliform and *E. coli*), total nitrogen, nitrate, ammonium, total phosphorus, and soluble-reactive phosphorus concentrations in surface waters; 2) compare results to a) water quality regulatory benchmarks, b) recommended maximum nutrient concentrations, and c) estimates of nutrient background concentrations; and 3) examine relationships between water quality, environmental conditions, cattle grazing, and recreation. Nutrient concentrations observed throughout the grazing-recreation season were at least one order of magnitude below levels of ecological concern, and were similar to U.S. Environmental Protection Agency (USEPA) estimates for background water quality conditions in the region. The relative percentage of FIB regulatory benchmark exceedances widely varied under individual regional and national water quality standards. Relative to USEPA’s national *E. coli* FIB benchmarks–the most contemporary and relevant standards for this study–over 90% of the 743 samples collected were below recommended criteria values. FIB concentrations were significantly greater when stream flow was low or stagnant, water was turbid, and when cattle were actively observed at sampling. Recreation sites had the lowest mean FIB, total nitrogen, and soluble-reactive phosphorus concentrations, and there were no significant differences in FIB and nutrient concentrations between key grazing areas and non-concentrated use areas. Our results suggest cattle grazing, recreation, and provisioning of clean water can be compatible goals across these national forest lands.

## Introduction

Livestock grazing allotments on public lands managed by the United States Forest Service (USFS) provide critical forage supporting ranching enterprises and local economies [1]–[3]. Surface waters on public lands are used for human recreation and consumption, and serve as critical aquatic habitat. Concerns have been raised that microbial and nutrient pollution by livestock grazing on public lands degrades water quality, threatening human and ecological health [4]–[7]. Some of the contaminants of concern include fecal indicator bacteria (FIB), fecal coliform (FC) and *Escherichia coli* (*E. coli*), as well as nitrogen (N) and phosphorus (P). FIB are regulated in an attempt to safeguard public health from waterborne pathogens such as *Cryptosporidium parvum* and *E. coli* O157:H7 and human enteroviruses including adenoviruses and coliphages [8]. Concerns about elevated N and P concentrations in surface water stem from the potential for eutrophication of aquatic systems [9].

The USFS must balance the many resource use activities occurring on national forests (e.g., livestock grazing, recreation). National forests in the western United States support 1.8 million livestock annually, provisioning 6.1 million animal unit months (AUM) of forage supply allocated through 5,220 grazing permits held by private ranching enterprises [10]. In California (USFS Region 5), 500 active grazing allotments annually supply 408,000 AUM of forage to support 97,000 livestock across 3.2 million ha on 17 national forests. With an annual recreating population of over 26 million [11], California’s national forests are at the crossroad of a growing debate about the compatibility of livestock grazing with other activities (e.g., recreation) dependent upon clean, safe water.

There is a paucity of original research on water quality conditions on public grazing lands, and the conclusions of these reports are often inconsistent. For example, in California’s Sierra Nevada, Derlet and Carlson [6] found surface water samples collected below horse and cattle grazing areas on USFS-administered lands were more likely to have detectable *E. coli* than non-grazed sites in national parks. Derlet *et al.* [12] reported algal coverage, algal-*E. coli* associations, and detection of waterborne *E. coli* to be greatest at sites below cattle grazing and lowest below sites experiencing little to no human or cattle activity, with human recreation sites being intermediate. Also in the central Sierra Nevada, Myers and Whited [13] found FIB increased in surface waters below key grazing areas on USFS allotments following the arrival of cattle. However, Roche *et al.* [14] found no evidence of degradation of Yosemite toad breeding pool water quality in key grazing areas on three allotments in the Sierra National Forest of central California. Examining land-use and water quality associations in watersheds throughout the Cosumnes River Basin, Ahearn *et al.* [15] also reported water quality conditions in upper forested watersheds, which include USFS grazing allotments, to be well below levels of ecological concern.

The purpose of this study was to quantify microbial pollutant and nutrient concentrations during the summer cattle grazing and recreation season on 12 representative allotments across 5 national forests in northern California. Specific objectives were to 1) quantify FC, *E. coli*, total nitrogen, nitrate, ammonium, total phosphorus, and soluble-reactive phosphate concentrations in surface waters; 2) compare these results to a) water quality regulatory benchmarks, b) maximum nutrient concentrations recommended to avoid eutrophication, and c) estimates of nutrient background concentrations for this region; and 3) examine relationships between water quality, environmental conditions, and cattle grazing and recreation (i.e., resource uses).

## Methods

### Ethics Statement

Permission for site access was granted by the US Forest Service, and no permits were required.

### Study Area

**Figure 1:**
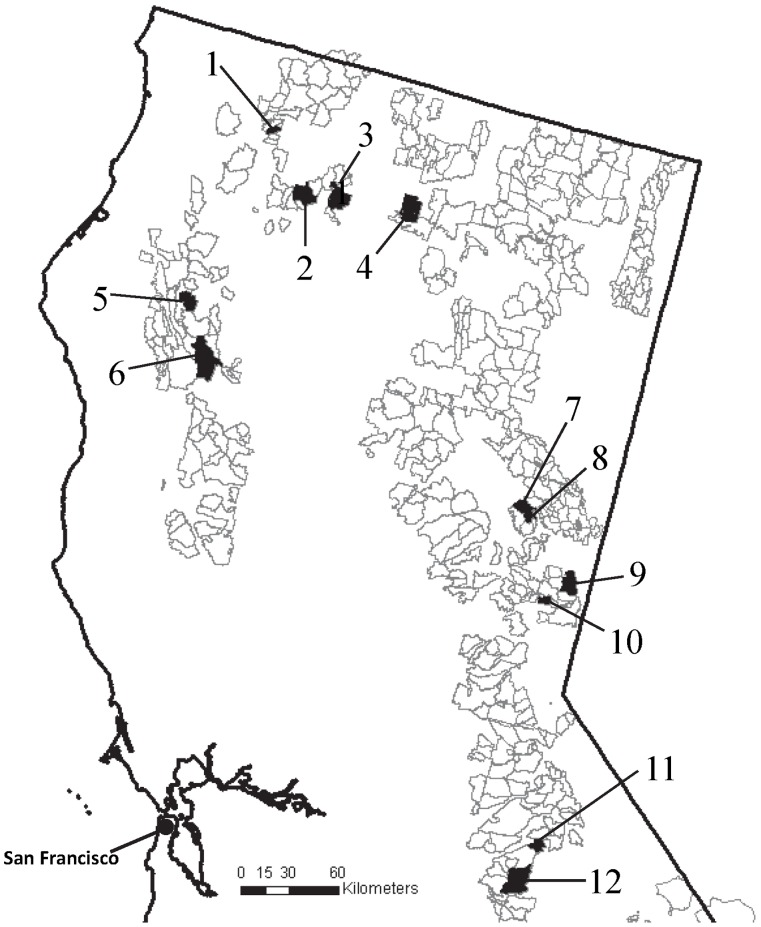
The 12 U.S. Forest Service grazing allotments (shaded polygons) in northern California enrolled in this cross-sectional longitudinal study of stream water quality between June and November 2011. Unshaded polygons are other U.S. Forest Service grazing allotments in the study area.

This cross sectional, longitudinal water quality survey was completed across 12 grazing allotments on USFS-managed public lands in northern California, USA (Fig. 1). Allotments were selected to represent the diversity of climate, soil, vegetation, water quality regulatory agencies, and resource use activities found across this landscape. The study area ranged from 41°40′ to 37°55′ N latitude and 123°30′ to 120°10′ W longitude, and included national forests in the Klamath, Coast, Cascade, and Sierra Nevada Mountain Ranges. Allotments were located on the Klamath (Allotments 1, 2), Shasta-Trinity (Allotments 3–6), Plumas (Allotments 7, 8), Tahoe (Allotments 9, 10), and Stanislaus (Allotments 11, 12) National Forests (Fig. 1). The study area totaled approximately1,300 km^2^ and elevation ranged from 207 to 3,016 m (Table S1). The prevailing climate is Mediterranean with cool, wet winters and warm, dry summers. The majority of precipitation falls as snow between December and April, with snow melt generally occurring between May and June. Soils in Allotments 1–2, 5–7, and 11 are dominated by Inceptisols; Allotments 3, 10, and 12 are dominated by Alfisols; Allotment 8 and 9 are dominated by Mollisols; and Allotment 4 is dominated by Andisols [16] (Table S1).

All allotments were located in mountainous watersheds with canopy cover of mesic and xeric forests ranging from 9 to 89 and 2 to 93% cover, respectively [17]. Cooler mesic conifer forests were dominated by white fir (*Abies concolor*), red fir (*Abies magnifica*), and Douglas fir (*Pseudotsuga menziesii*). The relatively drier xeric conifer forests were dominated by ponderosa pine (*Pinus ponderosa*) and Jeffrey pine (*Pinus jeffreyi*). Montane hardwood and shrub cover ranged from 0 to 20%, and grass and forb cover from 1 to 9%. Wet meadows and other riparian plant communities covered 1 to 5% of allotment areas, and were the primary forage source for cattle grazing in these allotments.

### Grazing Management

Cattle grazing management strategies on the study allotments reflect those widely found on western public grazing lands, such as those reviewed in Delcurto et al. [18] and George et al. [19]. Study allotments were grazed with commercial beef cow-calf pairs during the June to November grazing-growing season, following allotment-specific management plans designed to achieve annual herbaceous forage use standards (Table S1). Herbaceous use standards are set as an annual management target to protect ecological condition and function of meadow and riparian sites [20], and vary by national forest, allotment, and meadow ecological conditions [21]–[27].

Cattle stocking densities ranged from 1 animal unit (∼450 kg cow with or without calf) per 18 ha to 1 animal unit per 447 ha (Table S1). Timing of grazing (turn on and turn off dates for cattle), duration of grazing season, and number of cattle are permitted by the USFS on an allotment-specific basis. Animal unit month (AUM) is the mass of forage required to sustain a single animal unit for a 30-day period, and is the standard metric of grazing pressure on USFS allotments.

Foraging, and thus spatial distribution of cattle feces and urine, is non-uniform across these allotments. Areas receiving relatively concentrated use by cattle are referred to as key grazing areas. Key grazing areas are often relatively small, stream-associated meadows and riparian areas that are preferentially grazed by cattle due to high forage quantity and quality and drinking water availability. For the most part, allotments are not cross-fenced to create pastures, which would improve grazing distribution. Where cross-fences exist, resulting pasture sizes are large (>2000 ha) with few pastures per allotment (<3).

### Sample Site Selection

Key grazing areas and concentrated recreation areas within 200 m of streams in each allotment were identified and enrolled in the study in collaboration with local USFS managers and forest stakeholders. Water sample collection sites were established in streams immediately above, beside, and/or below sites with each activity to characterize water quality associated with these activities. Recreational activities included developed and undeveloped campgrounds, swimming-bathing areas, and trailheads used by hikers and recreational horse riders (i.e., pack stock). Key grazing areas were meadows and riparian areas that cattle were known to graze and occupy frequently and/or for extended periods throughout the grazing season. Additional sites were established at perennial flow tributary confluences with no concentrated use activities, enabling us to objectively include comparison sites across allotments with no concentrated grazing and/or recreation. While cattle use was concentrated primarily in key grazing areas, cattle grazing could occur throughout each allotment; therefore, it was not possible to determine water quality conditions in the complete absence of cattle.

A total of 155 stream water sample collection sites were identified and sampled monthly throughout the 2011 summer grazing-recreation period. Sample collection sites per allotment ranged from 7 to 18, depending upon the number of key grazing and recreation areas identified, and number of tributary confluences (Table S1). Sixty-three percent of sample sites were associated with key grazing areas, 17% were associated with recreation activities, and 20% were tributary confluences with no concentrated use activities.

### Sample Collection and Analysis

In 2011, a total of 743 water samples were collected and analyzed during the June 1 through November 9 study period, which captured the period of overlapping cattle grazing and recreation activities across these allotments. On each allotment, sampling occurred monthly throughout the grazing-recreation season. All sites in an allotment were sampled on the same day. Total sample numbers per allotment ranged from 40 to 88 (Table S1).

At the time of sample collection, environmental conditions and/or resource use activities that may have affected water quality were recorded. Specifically, the following conditions were noted (yes/no): 1) stagnant-low stream flow (<2 liters per second); 2) turbid stream water; 3) recreation (i.e., swimming-bathing, camping, hiking, fishing, horse riding); 4) cattle; and 5) any activities (i.e., low stream flow, turbid water, precipitation, cattle, recreation users) observed that may affect water quality. If algae, periphyton, or other aquatic autotrophic organisms were present at high to moderate levels (>20% of substrate cover) at time of sampling, then these conditions were recorded.

A vertical, depth-integrated stream water collection was made at the stream channel thalweg [28]. Water was collected in sterilized, acid-washed one liter sample containers, which were immediately stored on ice. All samples were analyzed for FC and *E. coli* within 8 hours of field collection. A 250 ml subsample was taken from each sample, frozen within 24 hours of collection, and processed for nutrient concentrations within 28 days of field collection. FC and *E. coli* concentrations as colony forming units (cfu) per 100 ml of water sample were determined by direct one step membrane filtration (0.45 µm nominal porosity filter) and incubation (44.5°C, 22–24 hours) on selective agar following standard method SM9222D [29]. Difco mFC Agar (Becton, Dickinson and Company, Spars, MD, USA) and CHROMagar *E. coli* (ChromAgar, Paris, France) were used for FC and *E. coli*, respectively. Total N (TN) and total phosphorus (TP) were measured after persulfate digestion of non-filtered subsamples following Yu *et al.* [*30*] and standard method SM4500-P.D [29], respectively. Concentrations of nitrate (NO_3_-N), ammonium (NH_4_-N), and soluble-reactive phosphorus (PO_4_-P) were determined from filtered (0.45 µm nominal porosity filter) subsamples following Doane and Horwath [31], Verdouw *et al.* [32], and Eaton *et al.* [29], respectively. Minimum detection limits were ∼10 µg L^−1^ for TN, NH_4_-N, and NO_3_-N and ∼5 µg L^−1^ for TP and PO_4_-P. Organic nitrogen (ON) was calculated as *TN* – [*NO_3_-N*+*NH_4_-N*], and non-soluble-reactive PO_4_-P was calculated as *TP – PO_4_-P*. Laboratory quality control included replicates, spikes, reference materials, control limits, criteria for rejection, and data validation methods [33].

### Data Analysis and Interpretation

Descriptive statistics were calculated for the overall dataset as well as by 1) key grazing areas, recreation areas, and sample sites with no concentrated resource use; 2) activity observed at time of sample collection; 3) and month. Results were compared to numerous FIB benchmark concentrations used in the formulation of contemporary microbial water quality standards, maximum nutrient concentrations recommended to avoid eutrophication, and background nutrient concentration estimates for surface waters across the study area. The United States Environmental Protection Agency (USEPA) nationally recommends and has provided guidance on *E. coli* FIB-based standards ranging from 100 to 410 cfu 100 ml^−1^, dependent upon selected illness rate benchmarks and frequency of sample collection over a 30 day period [34]. The study area falls within the jurisdiction of three semi-autonomous California Regional Water Quality Control Boards (RWQCBs), each of which has established enforceable standards based on FC benchmarks [35]–[37] ranging from 20 to 400 cfu 100 ml^−1^. We report study results relative to each of these benchmarks to allow for comparisons to the various national and regional policies. For our study, which is based on monthly monitoring of multiple land-use activity types and environmental conditions across a broad regional scale (spanning approximately1,300 km^2^), the most relevant and contemporary comparisons are the national U.S. Environmental Protection Agency (USEPA) *E. coli* single sample-based [8], [34] standards of 190 cfu 100 ml^−1^ (estimated illness rate of 32 per 1,000 primary contact recreators) and 235 cfu 100 ml^−1^ (estimated illness rate of 36 per 1,000 primary contact recreators).

General recommendations for maximum concentrations to prevent eutrophication of streams and rivers are 300, 100, and 50 µg L^−1^ for NO_3_-N, TP, and PO_4_-P, respectively [38]–[42]. The study area is within three USEPA Level III Sub-Ecoregions (5, 9, and 78), and estimated background concentrations for TN, NO_3_-N, and TP in these sub-regions range from 60 to 530, 5 to 40, and 9 to 32 µg L^−1^, respectively [43].

At the sample site-scale, we used bivariate generalized linear mixed effects models (GLMMs) and zero-inflated count models to test for mean FIB and nutrient concentration (dependent variables were fecal coliform, *E. coli*, TN, NO_3_-N, NH_4_-N, TP, and PO_4_-P) differences between 1) key grazing areas, recreation areas, and sample sites with no concentrated resource use; and 2) occurrence of stagnant-low stream flow, turbid stream water, cattle, and recreation at the time of sample collection. We used GLMMs to analyze dependent variables with overdisperison (i.e., greater variance than expected) (fecal coliform, *E. coli*, TN) using the Poisson probability distribution function with robust standard errors [44]. For the GLMMs, we specified allotment identity and sample site identity as sequential random effects to account for hierarchical nesting and repeated measures [44], [45]. Data with evidence of both overdispersion and zero-inflation can be produced by either unobserved heterogeneity or by processes that involve different mechanisms generating zero and nonzero counts [46]–[48]. For dependent variables with apparent overdispersion and zero-inflation (>25% zeros; NO_3_-N, NH_4_-N, TP, and PO_4_-P), we used likelihood ratio tests to evaluate relative fits of zero-inflated negative binomial versus zero-inflated Poisson models [46]–[48]; we used simple Vuong tests [49] to evaluate relative fits of zero-inflated versus standard count models; and we used either likelihood ratio tests or Akaike Information Criterion (AIC), as appropriate, to compare relative fits between negative binomial and Poisson models. To account for the within-cluster correlation due to repeated measures, we specified sample site identity as a clustering variable in the final models to obtain robust variance estimates [50].

We also examined allotment-scale relationships of FIB and nutrient concentrations with environmental conditions and grazing management. We used bivariate zero-truncated count models to test associations between mean allotment values of response variables (fecal coliform, *E. coli*, TN, NO_3_-N, NH_4_-N, TP, and PO_4_-P; mean of all samples collected for each allotment) and cattle grazing duration, animal unit months (AUM) of grazing, cattle density as cow-calf pairs 100 ha^−1^, mean allotment elevation, and 2011–2012 water year precipitation [42] (independent variables). We used likelihood ratio tests to compare Poisson and negative binomial models [48]. For all analyses, when multiple response variables were predicted with the same independent variables, we interpreted significance levels using Bonferroni corrections to safeguard against Type I errors. Bonferroni adjusted p-values were considered significant at 0.0071 (dividing *P = *0.05 by the 7 water quality indicators tested) and 0.0014 (dividing *P = *0.01 by the 7 water quality indicators tested). All statistical analyses were conducted in Stata/SE 11.1 [48].

## Results

### Surface Water Quality and Weather Conditions Observed during Study

**Figure 2:**
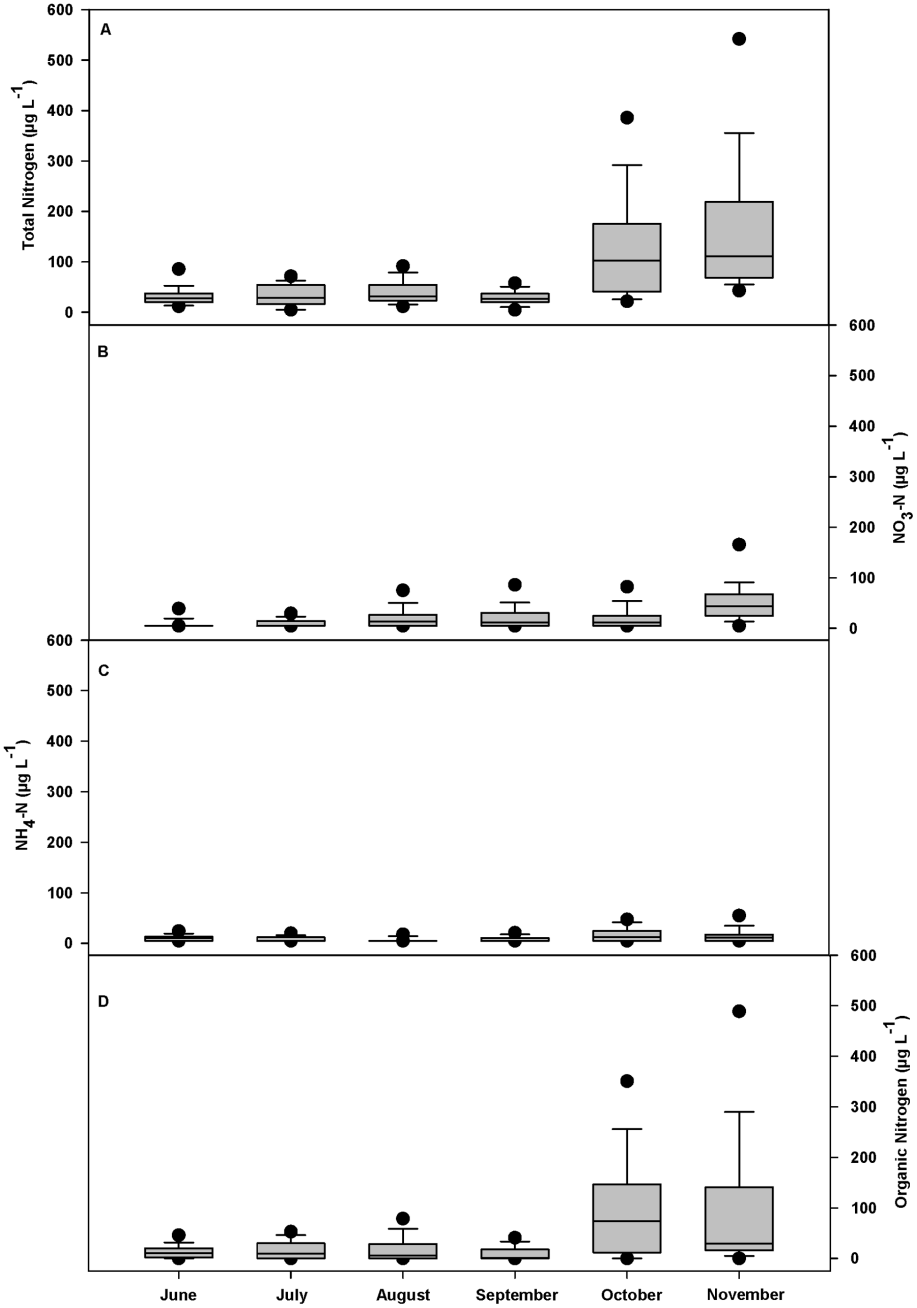
Overall monthly nitrogen concentrations for 743 stream water samples collected from 155 sample sites across 12 U.S. Forest Service grazing allotments in northern California enrolled in this cross-sectional longitudinal study between June and November 2011. (A) Total nitrogen, (B) nitrate (NO_3_-N), and (C) ammonium (NH_4_-N) were measured directly. (D) Organic nitrogen represents the difference between total nitrogen and NO_3_-N plus NH_4_-N. Bottom and top of shaded box are the 25^th^ and 75^th^ percentile of data, horizontal line within shaded box is median value, ends of vertical lines are 10^th^ and 90^th^ percentiles of data, and black dots are 5^th^ and 95^th^ percentiles of data. June *n* = 135; July *n* = 150; August *n* = 178; September *n* = 120; October *n* = 127; November *n = *33.

**Figure 3:**
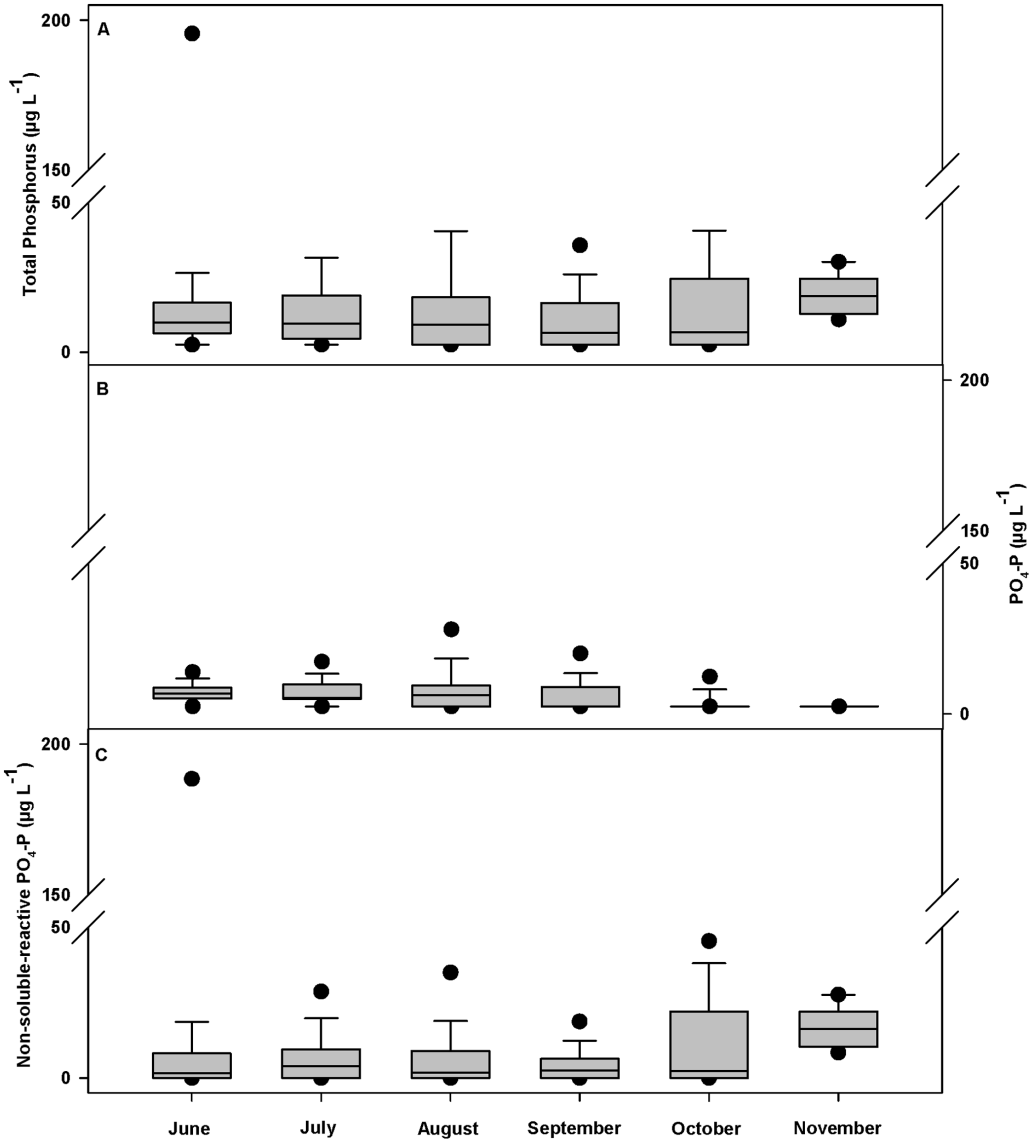
Overall monthly phosphorus concentrations for 743 stream water samples collected from 155 sample sites across 12 U.S. Forest Service grazing allotments in California enrolled in this cross-sectional longitudinal study between June and November 2011. (A) Total phosphorus (B) and soluble-reactive phosphorus (PO_4_-P) were measured directly. (C) Non-soluble-reactive phosphorus represents the difference between total phosphorus (measured on unfiltered sample and treated with digesting agent) and soluble-reactive phosphorus. Bottom and top of shaded box are the 25^th^ and 75^th^ percentile of data, horizontal line within shaded box is median value, ends of vertical lines are 10^th^ and 90^th^ percentiles of data, and black dots are 5^th^ and 95^th^ percentiles of data. June *n* = 135; July *n* = 150; August *n* = 178; September *n* = 120; October *n* = 127; November *n = *33.

**Figure 4:**
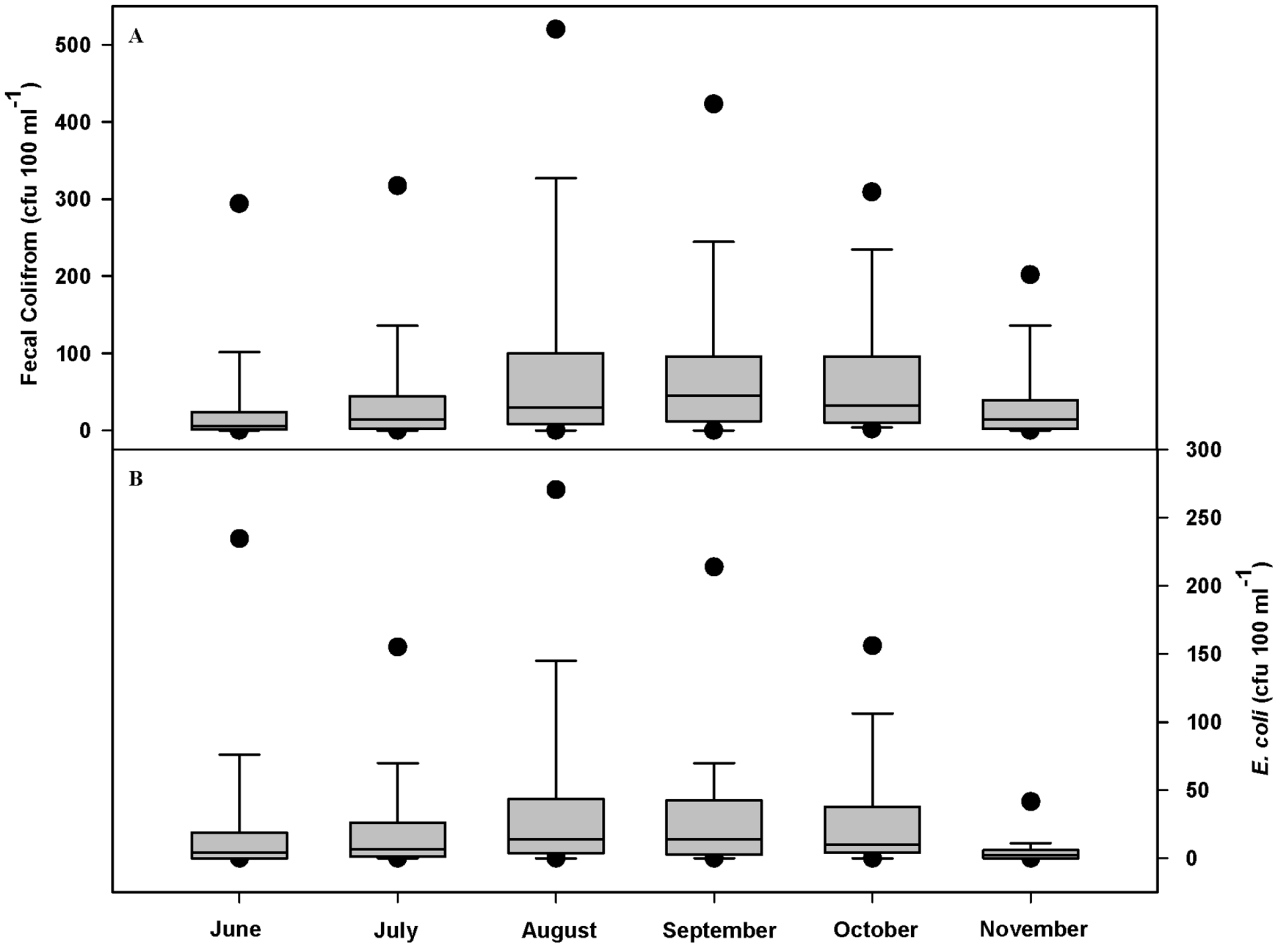
Overall monthly (A) fecal coliform and (B)*E.* *coli* concentrations for 743 stream water samples collected from 155 sample sites across 12 U.S. Forest Service grazing allotments in northern California enrolled in this cross-sectional longitudinal study between June and November 2011. Bottom and top of shaded box are the 25^th^ and 75^th^ percentile of data, horizontal line within shaded box is median value, ends of vertical lines are 10^th^ and 90^th^ percentiles of data, and black dots are 5^th^ and 95^th^ percentiles of data. June *n* = 135; July *n* = 150; August *n* = 178; September *n* = 120; October *n* = 127; November *n = *33.

Precipitation during the 2010–11 water year ranged from 88 to 173% of the 30-year mean annual precipitation for each allotment, with 11 of 12 allotments receiving over 100% of mean annual precipitation (Table S1). Overall, nutrient concentrations were low across the study area (Table 1). With the exception of TN, over 32% of samples were below minimum detection limits for all nutrients (<10 µg N L^−1^ and <5 µg P L^−1^). Nitrogen concentrations increased in October and November with the onset of fall rains (Fig. 2), and phosphorus concentrations showed no seasonal patterns (data not shown). The sum of NO_3_-N and NH_4_-N concentrations was lower than organic N (*TN* – [*NO_3_-N*+*NH_4_-N*]) concentrations throughout the sampling season (Fig. 2), suggesting that the majority of nitrogen was in organic forms. Additionally, PO_4_-P concentrations were much lower than TP (Table 1; Fig. 3), suggesting that the majority of phosphorus was either organic or inorganic P adsorbed to suspended sediments. Mean and maximum FC and *E. coli* concentrations per allotment ranged from 30 to 255 and 17 to 151 CFU 100 ml^−1^, and from 248 to 3,460 and 74 to 1,920, respectively (Table S2). FIB concentrations were highest from August through October (Fig. 4).

**Table 1:** Concentrations of total nitrogen (TN), nitrate (NO_3_-N), ammonium (NH_4_-N), total phosphorus (TP), and phosphate (PO_4_-P) for 743 stream water samples collected across 155 sample sites on 12 U.S. Forest Service grazing allotments in northern California.

Nutrient	Mean^a^ (µg L^−1^)	Median (µg L^−1^)	Maximum (µg L^−1^)	Below Detection^b^ (%)	Eutrophication^c^ (µg L^−1^)	Background^d^ (µg L^−1^)
TN	58±2.7	33	675	5	–	60–530
NO_3_-N	19±0.9	5	221	51	300	5–40
NH_4_-N	11±0.4	5	146	61	–	–
TP	21±2.8	9	1321	32	100	9–32
PO_4_-P	7±0.3	5	83	40	50	–

Published estimates of concentrations of general concern for eutrophication of stream water, and estimates of background concentrations for the study area are provided for context.

a The ‘±’ indicates 1 standard error of the mean.

b Percentage of samples below minimum analytical detection limit. Limits were 10 µg L^−1^ for nitrogen and 5 µg L^−1^ for phosphorous. Observations below detection limit were set to one half detection limit (5 µg L^−1^ for nitrogen and 2.5 µg L^−1^ for phosphorus) for calculation of mean and median concentrations.

c Concentrations if exceeded indicate potential for eutrophication of streams [38]–[42].

d Estimated range of background concentrations for the three U.S. Environmental Protection Agency Level III sub-ecoregions (5, 9, 78) included in the study [43].

### Nutrient and FIB Concentrations Relative to Water Quality Benchmarks

Mean and median NO_3_-N, TP, and PO_4_-P concentrations were at least one order of magnitude below nutrient concentrations recommended to avoid eutrophication (Table 1). No samples exceeded the NO_3_-N maximum recommendation (Table 1). Overall, less than 2% of samples exceeded eutrophication benchmarks (Table 2), and less than 8% of sites exceeded these benchmarks at least once (Table 3). Mean and median TN, NO_3_-N, and TP concentrations were at or below estimated background concentrations for the study area (Table 1). The percentage of all samples (Table 2) exceeding FIB benchmarks ranged from 50% (benchmark FC = 20 cfu 100 ml^−1^) to 1% (benchmark *E. coli* = 410 cfu 100 ml^−1^), while the percentage of sites (Table 3) that exceeded a FIB benchmark at least once ranged from 83% (benchmark FC = 20 cfu 100 ml^−1^) to 6% (benchmark *E. coli* = 410 cfu 100 ml^−1^).

**Table 2:** Percentage of 743 stream water samples collected across 155 sample sites on 12 U.S. Forest Service grazing allotments in northern California which exceeded water quality benchmarks relevant to the study area, specifically, and the nation, broadly.

Benchmark	Overall(% of 743)	Key Grazing Area(% of 462)	Recreation Area(% of 125)	No Concentrated Use Activities(% of 156)
FC >20 cfu 100 ml^−1a^	50	48	46	58
FC >50 cfu 100 ml^−1b^	31	28	27	42
FC >200 cfu 100 ml^−1c^	10	10	6	13
FC >400 cfu 100 ml^−1d^	4	5	2	4
*E. coli* >100 cfu 100 ml^−1e^	9	8	7	11
*E. coli* >126 cfu 100 ml^−1f^	7	7	6	8
* *E. coli* >190 cfu 100 ml^−1g^	5	4	4	6
* *E. coli* >235 cfu 100 ml^−1h^	3	3	3	4
*E. coli* >320 cfu 100 ml^−1i^	2	2	2	2
*E. coli* >410 cfu 100 ml^−1j^	1	2	2	1
NO_3_-N >300 µg L^−1k^	0	0	0	0
TP>100 µg L^−1l^	2	2	2	<1
PO_4_-P>50 µg L^−1m^	<1	1	0	0

Results are reported for samples collected across all sample sites (overall) as well as for samples collected at sample sites monitored to characterize specific resource use activities across the allotments.

* Indicates the most relevant and contemporary standards for this study.

a Fecal coliform (FC) benchmark designated by Lahontan Regional Water Quality Control Board (LRWQCB) (based on geometric mean (GM) of samples collected over a 30-day interval) [36].

b FC benchmark designated by North Coast Regional Water Quality Control Board (NCRWQCB) (based on a median of samples collected over a 30-day interval) [37].

c FC benchmark designated by Central Valley Regional Water Quality Control Board (CVRWQCB) (based on GM of samples collected over a 30-day interval) [35].

d FC benchmark designated by CVRWQCB and NCRWQCB (maximum threshold value not to be exceeded by more than 10% of samples over a 30-day interval) [35].

e *E. coli* benchmark designated by U.S. Environmental Protection Agency (USEPA) [34] for an estimated illness rate of 32 per 1,000 primary contact recreators (based on GM of samples collected over a 30-day interval).

f *E. coli* benchmark designated by USEPA [34] for an estimated illness rate of 36 per 1,000 primary contact recreators (based on GM of samples collected over a 30-day interval).

g *E. coli* benchmark designated by USEPA [34] for an estimated illness rate of 32 per 1,000 primary contact recreators (for a single grab sample, approximates the 75th percentile of a water quality distribution based on desired GM).

h *E. coli* benchmark designated by USEPA [34] for an estimated illness rate of 36 per 1,000 primary contact recreators (for a single grab sample, approximates the 75th percentile of a water quality distribution based on desired GM).^i^ *E. coli* benchmark designated by USEPA [34] for an estimated illness rate of 32 per 1,000 primary contact recreators (approximates the 90th percentile of a water quality distribution based on desired GM).

j *E. coli* benchmark designated by USEPA [34] for an estimated illness rate of 36 per 1,000 primary contact recreators (approximates the 90th percentile of a water quality distribution based on desired GM).^k^ Maximum concentrations of nitrate as nitrogen (NO_3_-N) recommended by USEPA [38], [39].

l Maximum concentrations of total phosphorus (TP) recommended by USEPA [39], [40].

m Maximum concentrations of phosphate as phosphorus (PO_4_-P) recommended by USEPA [39], [41].

**Table 3:** Percentage of 155 stream water sample sites on 12 U.S. Forest Service grazing allotments in northern California which had at least one exceedance of water quality benchmarks relevant to the study area, specifically, and the nation, broadly.

Benchmark	Overall(% of 155)	Key Grazing Area(% of 97)	Recreation Area(% of 27)	No Concentrated Use Activities(% of 31)
FC >20 cfu 100 ml^−1a^	83	82	81	87
FC >50 cfu 100 ml^−1b^	65	61	63	81
FC >200 cfu 100 ml^−1c^	34	36	22	39
FC >400 cfu 100 ml^−1d^	18	20	11	19
*E. coli* >100 cfu 100 ml^−1e^	29	31	22	29
*E. coli* >126 cfu 100 ml^−1f^	25	28	19	23
*E. coli >190 cfu 100 ml^−1g^	17	16	15	19
*E. coli >235 cfu 100 ml^−1h^	14	13	11	16
*E. coli* >320 cfu 100 ml^−1i^	8	6	11	10
*E. coli* >410 cfu 100 ml^−1j^	6	6	7	3
NO_3_-N >300 µg L^−1k^	0	0	0	0
TP>100 µg L^−1l^	8	10	7	3
PO_4_-P>50 µg L^−1m^	2	3	0	0

Results are reported for all sample sites (overall) as well as for sample sites monitored to characterize specific resource use activities across the allotments. *Indicates the most relevant and contemporary standards for this study.

a Fecal coliform (FC) benchmark designated by Lahontan Regional Water Quality Control Board (LRWQCB) (based on geometric mean (GM) of samples collected over a 30-day interval) [36].

b FC benchmark designated by North Coast Regional Water Quality Control Board (NCRWQCB) (based on a median of samples collected over a 30-day interval) [37].

c FC benchmark designated by Central Valley Regional Water Quality Control Board (CVRWQCB) (based on GM of samples collected over a 30-day interval) [35].

d FC benchmark designated by CVRWQCB and NCRWQCB (maximum threshold value not to be exceeded by more than 10% of samples over a 30-day interval) [35].

e *E. coli* benchmark designated by U.S. Environmental Protection Agency (USEPA) [34] for an estimated illness rate of 32 per 1,000 primary contact recreators (based on GM of samples collected over a 30-day interval).

f *E. coli* benchmark designated by USEPA [34] for an estimated illness rate of 36 per 1,000 primary contact recreators (based on GM of samples collected over a 30-day interval).

g *E. coli* benchmark designated by USEPA [34] for an estimated illness rate of 32 per 1,000 primary contact recreators (for a single grab sample, approximates the 75th percentile of a water quality distribution based on desired GM).

h *E. coli* benchmark designated by USEPA [34] for an estimated illness rate of 36 per 1,000 primary contact recreators (for a single grab sample, approximates the 75th percentile of a water quality distribution based on desired GM).^i^ *E. coli* benchmark designated by USEPA [34] for an estimated illness rate of 32 per 1,000 primary contact recreators (approximates the 90th percentile of a water quality distribution based on desired GM).

j *E. coli* benchmark designated by USEPA [34] for an estimated illness rate of 36 per 1,000 primary contact recreators (approximates the 90th percentile of a water quality distribution based on desired GM).^k^ Maximum concentrations of nitrate as nitrogen (NO_3_-N) recommended by USEPA [38], [39].

l Maximum concentrations of total phosphorus (TP) recommended by USEPA [39], [40].

m Maximum concentrations of phosphate as phosphorus (PO_4_-P) recommended by USEPA [39], [41].

### Nutrient and FIB Concentrations Relative to Grazing, Recreation, and Field Observations

Nutrient concentrations were at or below background levels, and only 0–10% of sites within each resource use activity category (i.e., key grazing areas, recreation areas, and non-concentrated use activities) had at least one nutrient benchmark exceedance (Table 3). The relative percentage of samples and sites exceeding FIB benchmarks for key grazing areas, recreation areas, and non-concentrated use areas varied by the individual benchmarks (Tables 2 and 3).

We found significantly (*P*<0.002) lower FC, *E. coli*, TN and PO_4_-P concentrations at recreation areas than at key grazing areas and areas with no concentrated use activities (Table 4). Mean NO_3_-N concentrations were also significantly lower (*P*<0.001) at recreation sites than at areas with no concentrated use activity; however, it is important to note that all nutrient concentrations were at or below background levels (Table 1), and none of the sites sampled ever exceeded the maximum recommended NO_3_-N concentrations during the study (Tables 3).

**Table 4:** Mean concentrations for fecal coliform (FC) and*E. coli*, total nitrogen (TN), nitrate as nitrogen (NO_3_-N), ammonium as nitrogen (NH_4_-N), total phosphorus (TP), and phosphate as phosphorus (PO_4_-P) for 743 total stream water samples collected across 155 sample locations on 12 U.S. Forest Service grazing allotments in northern California.

	Key Grazing Area	Recreation Area	No Concentrated Use Activities
	(462 samples)	(125 samples)	(156 samples)
FC (cfu 100 ml^−1^)	87±12 a	55±9 b	90±12 a
*E. coli* (cfu 100 ml^−1^)	42±6 a	29±7 b	43±8 a
Total N (µg L^−1^)	61±4 a	38±3 b	64±6 a
NO3-N (µg L^−1^)	17±1 ab	16±1 a	25±2 b
NH4-N (µg L^−1^)	11±0.6 a	10±1 a	10±0.7 a
Total P (µg L^−1^)	24±4 a	14±4 a	17±2 a
PO4-P (µg L^−1^)	7±0.3 a	5±0.2 b	8±0.6 a

Results reported are mean concentration for each resource use activity category. The ‘±’ indicates 1 standard error of the mean. Different lower case letters indicate significant (*P*<0.05 with Bonferroni-correction for multiple comparisons) differences between resource use activity categories.

Relative to conditions at time of sample collection, FC, *E. coli*, and PO_4_-P concentrations were significantly (*P*<0.0071) higher when stream flow was low or stagnant, stream water was turbid, and when cattle were actively observed (Table 5). TP concentrations were also significantly higher (*P*<0.001) under turbid water conditions. *E. coli*, TN, NH_4_-N, and PO_4_-P concentrations were significantly lower (*P*<0.006) when recreation activities were observed at time of sampling, compared to sample events when recreation was not occurring (Table 5). Occurrence of high to moderate cover (>20% of substrate cover) of algae, periphyton, and other aquatic organisms at time of sampling was low (<2% of samples).

**Table 5:** Mean concentrations for fecal coliform (FC) and*E. coli*, total nitrogen (TN), nitrate as nitrogen (NO_3_-N), ammonium as nitrogen (NH_4_-N), total phosphorus (TP), and phosphate as phosphorus (PO_4_-P) for 743 total stream water samples collected across 155 sample locations on 12 U.S. Forest Service grazing allotments in northern California.

	Low Stream Flow^a^		Turbid Water^b^		Cattle Present^c^		Recreation^d^		Activities Observed^e^	
	Yes	No	Yes	No	Yes	No	Yes	No	Yes	No
No. Occurrences	51	692	37	706	130	613	28	715	341	402
FC (cfu 100 ml^−1^)	216±67^**^	72±7	212±64^**^	76±8	205±39^**^	56±5	36±13	84±8	115±16^**^	54±6
*E. coli* (cfu 100 ml^−1^)	114±45^*^	35±3	142±56^**^	35±3	115±21^**^	24±3	14±5^*^	41±4	61±9^*^	23±3
Total N (µg L^−1^)	87±16	55±3	95±12	56±3	44±4	60±3	27±3^**^	59±3	48±3	65±4
NO_3_-N (µg L^−1^)	17±3	19±1	19±1	16±3	19±2	18±1	16±3	19±1	17±1	20±1
NH_4_-N (µg L^−1^)	15±3	10±0.4	10±0.4	13±2	9±1	11±0.5	7±0.7^**^	11±0.4	10±0.6	11±0.5
Total P (µg L^−1^)	30±5	20±3	107±37^**^	16±2	20±3	21±3	10±2	21±3	27±6^*^	15±1
PO_4_-P (µg L^−1^)	13±2^**^	7±0.2	11±2^**^	7±0.2	10±1^*^	6±0.2	6±0.5^**^	7±0.3	7±0.5	5±0.3

Results are reported by category of field observation of resource use activities and environmental conditions observed at the time of sample collection. The ‘±’ indicates 1 standard error of the mean, * indicates different at *P*<0.05 (Bonferroni-adjusted), and ** indicates different at *P*<0.01 (Bonferroni-adjusted).

a Stagnant or low stream flow (<2 liters per second).

b Stream water turbid.

c Cattle observed.

d Recreational activities only (i.e., no cattle present) observed.

e Any activities (low stream flow, turbid water, precipitation, cattle, or recreation) observed that potentially impact water quality.

### Allotment-scale Nutrient and FIB Concentrations Relative to Grazing Management and Environmental Conditions

Mean allotment-scale nutrient concentrations were not significantly related (at Bonferroni adjusted *P*<0.0071) to cattle density (TN: *P* = 0.3; NO_3_-N: *P* = 0.2; NH_4_-N: *P* = 0.2; TP: *P* = 0.3; PO_4_-P: *P* = 0.1), precipitation (TN: *P* = 0.09; NO_3_-N: *P* = 0.07; NH_4_-N: *P* = 0.73; TP: *P* = 0.3; PO_4_-P: *P* = 0.04), mean allotment elevation (TN: *P* = 0.02; NO_3_-N: *P* = 0.4; NH_4_-N: *P* = 0.07; TP: *P* = 0.5; PO_4_-P: *P* = 0.2), AUM (TN: *P* = 0.6; NO_3_-N: *P* = 0.5; NH_4_-N: *P* = 0.9; TP: *P* = 0.1; PO_4_-P: *P* = 0.6), or grazing duration (TN: *P* = 0.02; NO_3_-N: *P* = 0.5; NH_4_-N: *P* = 0.03; TP: *P* = 0.6; PO_4_-P: *P* = 0.6).

**Figure 5:**
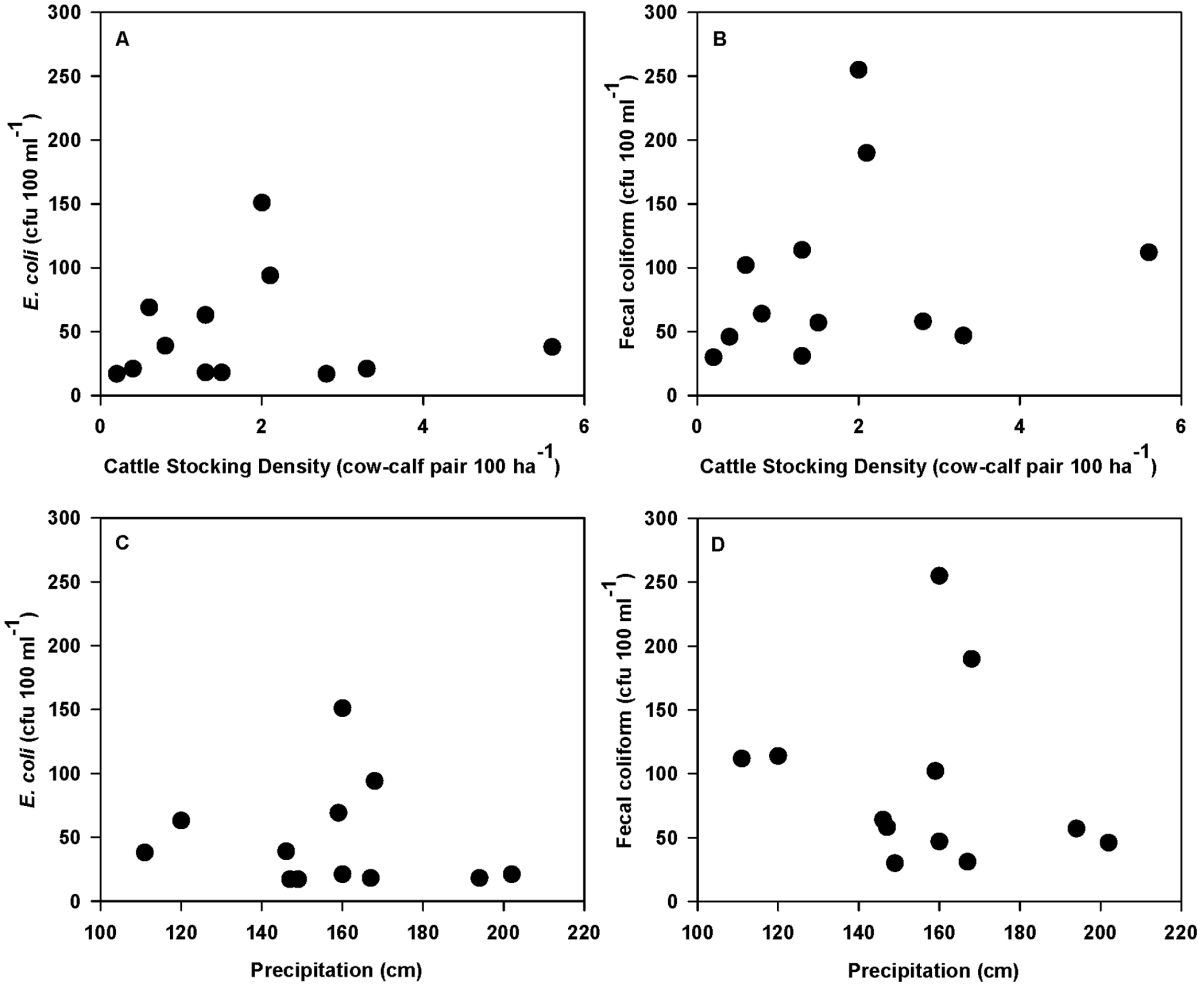
Trends in overall mean fecal indicator bacteria concentrations across sample sites during the June through November 2011sample period on 12 U.S. Forest Service grazing allotments in northern California enrolled in this cross-sectional longitudinal study. There were no significant relationships between allotment cattle stocking density and mean allotment concentrations of (A) *E. coli* (*P*>0.9) and (B) fecal coliform (*P*>0.3). During the study period, there were also no significant relationships between 2010–2011 water year precipitation and mean allotment concentrations of (C) *E. coli* (*P*>0.6) and (D) fecal coliform (*P*>0.5).

Mean allotment *E. coli* and FC concentrations showed increasing trends with increasing cattle densities and AUMs, and decreasing trends with increasing precipitation; however, these relationships were not statistically significant (*P*>0.2; Fig. 5). Mean allotment elevation (*P*>0.8), and cattle grazing duration (*P*>0.7) were also not correlated to mean allotment FIB concentrations (data not shown).

## Discussion

### Nutrient Conditions Relative to Water Quality Benchmarks

Mean and median nutrient concentrations observed across this grazed landscape were well below eutrophication benchmarks and background estimates (Table 1) [38]–[43]. Observed peak values in nitrogen and phosphorus concentrations were largely organic (or inorganic P adsorbed to suspended sediments) (Figs. 2 and 3), which are not considered readily available to stimulate primary production and eutrophication [39], [51]. These results do not support concerns that excessive nutrient pollution is degrading surface waters on these USFS grazing allotments [4], [12]. Our nutrient results are consistent with other examinations of surface water quality in similarly grazed landscapes. In the Sierra Nevada, Roche *et al.* [14] found nutrient concentrations of surface waters within key cattle grazing areas (mountain meadows) to be at least an order of magnitude below levels of ecological or biological concern for sensitive amphibians. On the Wallowa-Whitman National Forest in northeastern Oregon, Adams *et al.* [52] also reported nutrient levels to be at or below minimum detection levels in surface waters at key grazing areas.

Our results also agree with other studies of nutrient dynamics in the study area [53], [54]. Headwater streams, such as those draining the study allotments, typically make up 85% of total basin scale drainage network length, have high morphological complexity, and high surface to volume ratios–which make them particularly effective at nutrient processing and retention [55]. Leonard *et al* [54] found that drainages in the western Tahoe Basin recovering from past disturbances and undergoing secondary succession tend to act as sinks for nutrients. Several studies have reported nutrient limitations across montane and subalpine systems resulting in low riverine nutrient export [56].

### FIB Concentrations Relative to Water Quality Benchmarks

Overall mean and median *E. coli* were 40 and 8 cfu 100 ml^−1^, and mean and median FC were 82 and 21 cfu 100 ml^−1^ (Table S2)– indicating that the nationally recommended *E. coli* FIB-based benchmarks would be broadly met, and that the more restrictive, FC FIB-based regional water quality benchmarks would be commonly exceeded across the study region. Clearly, assessments of microbial water quality and human health risks are dependent upon which FIB benchmarks are used for evaluation (Tables 2 and 3).

The scientific and policy communities are currently evaluating the utility of, and guidance for, FIB-based water quality objective effectiveness for safe-guarding recreational waters. As reviewed in Field and Samadpour [8], *E. coli* and FC are not always ideal indicators of fecal contamination and risk to human health from microbial pathogens. Poor correlations between bacterial indicators and pathogens such as *Salmonella* spp., *Giardia* spp., *Cryptosporidium* spp., and human viruses undermine the utility of these bacteria as *indicators* of pathogen occurrence and human health risk [8]. The ability of FIB to establish extra-intestinal, non-animal, non-human associated environmental strains and to grow and reproduce in water, soil sediments, algal wrack, and plant cavities also erodes their utility as *indicators* of animal or human fecal contamination [8]. Citing scientific advancements in the past two decades, the USEPA now recommends adoption of an indicator *E. coli* water quality objective as an improvement over previously used general indicators, including FC [34]. This guidance is based, in part, on *E. coli* exhibiting relatively fewer of the fecal indicator bacteria utility issues listed above, and on evidence that *E. coli* is a better predictor of gastro-intestinal illness than FC. Therefore, comparing our results to the most relevant and scientifically defensible *E. coli* FIB-based recommendations, 17% of all sites exceeded the 190 cfu 100 ml^−1^ benchmark, and 14% of all sites exceeded the 235 cfu 100 ml^−1^ benchmark [34]. This analysis, based on the best available science and USEPA guidance, clearly contrasts with the FC FIB-based interpretations currently in use by several regional regulatory programs, which suggest that as many as 83% of all sites in our study present potential human health risks.

### Temporal Patterns in Water Quality

We observed a marked increase in total nitrogen concentrations in October and November, driven primarily by increased organic nitrogen, and to a lesser extent NO_3_-N (Fig. 2). This coincided with the first rainfall-runoff events of fall that initiated flushing of solutes and particulates. The annual fall flush occurs subsequent to the summer drought and base flow period during which organic and inorganic nutrient compounds accumulate in soil and forest litter [54], [57]–[60]. The disparity between TN and inorganic nitrogen (NO_3_-N+NH_4_-N) indicates the majority of flushed nitrogen was either particulate or dissolved organic nitrogen (Fig. 2). Consequently, most of the nitrogen flushed was likely in a relatively biologically unavailable form [51], with limited risk (relative to inorganic forms) of stimulating primary production and eutrophication. However, in nitrogen limited systems, increased biological utilization of organic nitrogen can occur [61].

FIB concentrations were highest from August through October (Fig. 4), which coincides with the period of maximum number of cattle turned out (Table S1). There is clear evidence that FIB concentrations increase with the introduction of cattle into a landscape, and increase with increasing cattle numbers [62]–[65]. The observed seasonal pattern of peak FIB concentrations also tracks the progression of stream flow from high, cold spring snowmelt to low, warm late-summer base flow conditions. Warm, low-flow conditions have been associated with elevated FIB [66]–[68]. Across this region, stream water temperatures are at their annual maximum in August and stream flows are at their annual minimum in September [69], [70]. We observed stagnant-low flow conditions to be significantly associated with increased FIB concentrations (Table 5). It is likely that the seasonal peak of FIB concentrations is driven by timing of maximum annual cattle numbers, as well as optimal environmental conditions for growth and in-stream retention of both animal-derived and environmental bacteria (e.g., wildlife sources) [71]–[73]. Similar temporal trends in FIB concentrations have been observed in surface waters of Oregon, Wyoming, and Alaska [65], [74], [75].

### Water Quality, Grazing, Recreation, and Environmental Conditions

Mean FIB concentrations at key grazing and non-concentrated use areas were higher than recreation sites, but did not exceed USEPA *E. coli* FIB-based benchmarks (Table 4). Mean FIB concentrations for all resource use activity categories exceeded the most restrictive regional FC FIB-based benchmarks of 20 and 50 cfu 100 ml^−1^. *E. coli* FIB-based benchmark comparisons were generally comparable across sites, with recreation sites exhibiting overall lower numbers of exceedances; however, the different FC FIB-based benchmark comparisons indicated inconsistent results for water quality conditions across sites (Table 3). Similar to other surveys in the region [6], [12], [13], FIB concentrations were significantly greater when cattle were present at time of sample collection (Table 5). Tiedemann *et al.* [65] observed the same trend, with higher stream water FC concentrations on forested watersheds experiencing relatively intensive cattle grazing compared to ungrazed watersheds. Gary *et al.* [63] found grazing to have relatively minor impacts on water quality, though a statistically significant increase in stream water FC concentrations was induced at a relatively high stocking rate.

Mean allotment FIB concentrations showed apparent increasing trends with greater cattle densities (Fig. 5A and 5B); however, these allotment-level relationships were not statistically significant. Decreasing cattle density lowers fecal-microbial pollutant loading [76], which has been shown to reduce FIB concentrations in runoff from grazed landscapes [77]. Decreasing cattle density may also reduce stream bed disturbance and re-suspension of FIB-sediment associations by cattle [78]–[82]. Attracted to streams for shade, water, and riparian forage, cattle have been shown to spend approximately 5% of their day within or adjacent to a stream [63], depositing about 1.5% of their total fecal matter within one meter of a stream [83]. In a comprehensive review, George et al. [19] found that management practices that reduce livestock densities, residence time, and fecal and urine deposition in streams and riparian areas can reduce nutrient and microbial pollutant loading of surface water.

Samples associated with turbid stream water at the time of sample collection had significantly higher mean FIB concentrations than samples associated with non-turbid conditions (Table 5). It has been well documented that stream sediments contain higher concentrations of FIB than overlying waters [78]–[80], [82], and that re-suspension of sediments in the water column by factors such as cattle disturbance or elevated stream flow is associated with elevated water column FIB concentrations [81]. FIB concentrations were also significantly higher under stagnant-low flow conditions (Table 5). Schnabel *et al.* [75] found a negative correlation between stream discharge and FIB concentrations at some sites, possibly due to the absence of a dilution effect under low flow conditions.

Although not statistically significant, we observed decreasing mean allotment FIB concentrations with greater precipitation during the 2010–2011 water-year (October 1 to September 30) (Fig. 5C and 5D). It is likely that precipitation during the 2010–2011 water-year is primarily reflecting snowpack, which supported higher than historical stream flow volumes during the study period. This potential relationship possibly reflects capacity of higher base flow volumes to dilute FIB concentrations. Lewis *et al.* [84] observed a similar negative correlation between surface runoff FC concentrations and annual cumulative precipitation on California coastal dairy pastures. Our observation that maximum FIB concentrations occurred under stagnant-low flow conditions (Table 5) also supports the potential for a negative relationship between FIB concentrations and annual precipitation.

Our results do not support previous concerns of widespread microbial water quality pollution across these grazed landscapes, as concluded in other surveys [6], [12], [13]. Although we did find apparent trends between cattle density and FIB concentrations (Figs. 5A and 5B) and significantly greater FIB concentrations when cattle were actively present, only 16% and 13% (Table 3) of key grazing areas (n = 97) exceeded the *E. coli* FIB-based benchmarks of 190 cfu 100 m^−1^ and 235 cfu 100 m^−1^, respectively. Only 5 and 3% of total samples collected exceeded the *E. coli* FIB-based benchmarks of 190 cfu 100 m^−1^ and 235 cfu 100 m^−1^, respectively (Table 2). In contrast, Derlet et al. [6] reported 60% and 53% of cattle grazing sites (n = 15) exceeded the 190 cfu 100 m^−1^ and 235 cfu 100 m^−1^ benchmarks, respectively. We also found no significant differences in FIB concentrations among key grazing areas and areas of no concentrated use activities (Table 4), which contrasts with previous work in the Sierra Nevada [6], [12]. Finally, in this landscape of mixed livestock grazing and recreational uses, we found FIB concentrations to be lowest at recreation sites, indicating that water recreation objectives can be broadly attained within these grazing allotments. There are three important distinctions that separate our study from previous work: 1) in reaching our conclusions, we compared our study results to regulatory and background water quality benchmarks, which are based on current and best available science and policy; 2) these co-occurring land-use activities were directly compared on the same land units managed by a single agency (USFS), as opposed to previous comparisons between these land-uses occurring on different management units administered by different agencies with very different land-use histories and policies (e.g., USFS and U.S. National Park Service); and 3) to date, this study is the most comprehensive water quality survey in existence for National Forest public grazing lands, including an assessment of seven water quality indicators at 155 sites across five National Forests.

### Conclusions

Nutrient concentrations observed across this extensively grazed landscape were at least one order of magnitude below levels of ecological concern, and were similar to USEPA estimates for background conditions in the region. Late season total nitrogen concentrations increased across all study allotments due to a first flush of organic nitrogen associated with onset of fall rainfall-runoff events, as is commonly observed in California’s Mediterranean climate. Similar to previous work, we found greater FIB concentrations when cattle were present; however, we did not find overall significant differences in FIB concentrations between key grazing areas and non-concentrated use areas, and all but the most restrictive, FC FIB-based regional water quality benchmarks were broadly met across the study region. Although many regional regulatory programs utilize the FC FIB-based standards, the USEPA clearly states–citing the best available science–*E. coli* are better indicators of fecal contamination and therefore provide a more accurate assessment of water quality conditions and human health risks. Throughout the study period, the USEPA recommended *E. coli* benchmarks of 190 and 235 cfu 100 ml^−1^ were met at over 83% of sites. These results suggest cattle grazing, recreation, and clean water can be compatible goals across these national forest lands.

## Supporting Information

Table S1 **Geographic characteristics, study year precipitation, cattle grazing management, and water quality sample collection sites and sample numbers for 12 U.S. Forest Service grazing allotments in northern California enrolled in this cross-sectional longitudinal study of stream water quality between June and November 2011.**
(DOCX)

Table S2 **Mean, median, and maximum fecal coliform (FC) and** ***E. coli*** **concentrations for 743 stream water samples collected across 155 sample sites on 12 U.S. Forest Service grazing allotments in northern California.** All concentrations are reported as colony forming units per 100 ml of sample water (cfu 100 ml^−1^).
(DOCX)

## References

[1] Gentner BJ, Tanaka JA (2002) Classifying federal public land grazing permittees. J Range Manage 55: 2–11.

[2] Huntsinger L, Forero LC, Sulak A (2010) Transhumance and pastoralist resilience in the Western United States. Pastoralism 1: 9–36.

[3] Sulak A, Huntsinger L (2002) The importance of federal grazing allotments to central Sierran oak woodland permittees: A first approximation. In: Standiford RB, editor. Fifth symposium on oak woodlands: Oaks in California’s changing landscape, PSW-GTR-184. Albany, California: USDA Forest Service Pacific Southwest Research Station. 43–51.

[4] Belsky AJ, Matzke A, Uselman S (1999) Survey of livestock influences on stream and riparian ecosystems in the western United States. J Soil Water Conserv 54: 419–431.

[5] Brunson MW, Steel BS (1996) Sources of variation in attitudes and beliefs about federal rangeland management. J Range Manage 49: 69–75.

[6] Derlet RW, Carlson JR (2006) Coliform bacteria in Sierra Nevada wilderness lakes and streams: What is the impact of backpackers, pack animals, and cattle? J Wilderness Med 17: 15–20. 10.1580/pr05-05.1 16538940

[7] Taylor FR, Gillman LA, Pedretti JW (1989) Impact of cattle on 2 isolated fish populations in Pahranagat Valley, Nevada. Great Basin Nat 49: 491–495.

[8] Field KG, Samadpour M (2007) Fecal source tracking, the indicator paradigm, and managing water quality. Water Res 41: 3517–3538. 17643471 10.1016/j.watres.2007.06.056

[9] Conley DJ, Paerl HW, Howarth RW, Boesch DF, Seitzinger SP, et al (2009) Controlling eutrophication: Nitrogen and phosphorus. Science 323: 1014–1015. 19229022 10.1126/science.1167755

[10] USFS (2011) Grazing statistical summary fiscal year 2009. Washington, D.C.: USDA Forest Service. 108 p.

[11] USFS (2012) National visitor use monitoring results - USDA Forest Service national summary report. Washington, D.C.: USDA Forest Service. 31 p.

[12] Derlet RW, Richards JR, Tanaka LL, Hayden C, Ger KA, et al (2012) Impact of summer cattle grazing on the Sierra Nevada watershed: Aquatic algae and bacteria. J Env Pub Health 2012: 1–7. 10.1155/2012/760108 PMC3312331 22505950

[13] Myers L, Whited B (2012) The impact of cattle grazing in high elevation Sierra Nevada mountain meadows over widely variable annual climatic conditions. J Env Protection 3: 823–837.

[14] Roche LM, Allen-Diaz B, Eastburn DJ, Tate KW (2012) Cattle grazing and Yosemite toad (*Bufo canorus* Camp) breeding habitat in Sierra Nevada meadows. Rangeland Ecol Manag 65: 56–65.

[15] Ahearn DS, Sheibley RW, Dahlgren RA, Anderson M, Johnson J, et al (2005) Land use and land cover influence on water quality in the last free-flowing river draining the western Sierra Nevada, California. J Hydrol 313: 234–247.

[16] Soil Survey Staff, Natural Resources Conservation Staff (2008) Soil Survey Geographic Database (SSURGO). Available: http://soils.usda.gov/survey/geography/ssurgo/. Accessed 2013 May 31.

[17] USFS (2009) CALVEG 2000, USDA Forest Service Pacific Southwest Region Remote Sensing Lab website. Available: http://www.fs.fed.us/r5/rsl/projects/gis/data/vegcovs/cvalley/EvegTile33A_00_07_100k_v2.html. Accessed 2013 May 31.

[18] Delcurto T, Porath M, Parsons CT, Morrison JA (2005) Management strategies for sustainable beef cattle grazing on forested rangelands in the Pacific Northwest. Rangeland Ecol Manag 58: 119–127.

[19] George MR, Jackson RD, Boyd CS, Tate KW (2011) A scientific assessment of the effectiveness of riparian management practices. In: Bridke DD, editor. Conservation benefits of rangeland practices: Assessment, recommendations, and knowledge gaps. Lawrence: Allen Press. 213–252.

[20] Clary WP, Leininger WC (2000) Stubble height as a tool for management of riparian areas. J Range Manage 53: 562–573.

[21] BLM (1999) Utilization studies and residual measurements. Denver: USDI Bureau of Land Management. 174 p.

[22] USFS (1988) Plumas National Forest land and resource management plan website. Available: http://www.fs.usda.gov/land/plumas/landmanagement. Accessed 2013 May 23.

[23] USFS (1990) Tahoe National Forest land and resource management plan website. Available: http://www.fs.usda.gov/detail/tahoe/landmanagement. Accessed 2013 May 23.

[24] USFS (1995) Shasta-Trinity National Forest land and resource management plan website. Available: http://www.fs.usda.gov/main/stnf/landmanagement/planning. Accessed 2013 May 23.

[25] USFS (2004) 2004 Sierra Nevada Forest plan amendment record of decision. Vallejo, California: USDA Forest Service. 72 p.

[26] USFS (2010) Klamath National Forest land and resource management plan website. Available: http://www.fs.usda.gov/main/klamath/landmanagement/planning. Accessed 2013 May 23.

[27] USFS (2010) Stanislaus National Forest land and resource management plan website. Available: http://www.fs.usda.gov/main/stanislaus/landmanagement/planning. Accessed 2013 May 23.

[28] Dahlgren RA, Tate KW, Ahearn DS (2004) Watershed scale, water quality monitoring – water sample collection. In: Down RD, Lehr JH, editors. Environmental instrumentation and analysis handbook. New York: John Wiley and Sons. 547–564.

[29] Eaton AD, Clesceri LS, Rice EW, Greenberg AE, Franson MAH (2005) Standard methods for the examination of water and wastewater: Centennial edition. Washington, D.C.: American Public Health Association. 1368 p.

[30] Yu ZS, Northup RR, Dahlgren RA (1994) Determination of dissolved organic nitrogen using persulfate oxidation and conductimetric quantification of nitrate-nitrogen. Commun Soil Sci Plan 25: 3161–3169.

[31] Doane TA, Horwath WR (2003) Spectrophotometric determination of nitrate with a single reagent. Anal Lett 36: 2713–2722.

[32] Verdouw H, Vanechteld CJA, Dekkers EMJ (1978) Ammonia determination based on indophenol formation with sodium salicylate. Water Res 12: 399–402.

[33] Puckett M (2002) Quality assurance management plan for the state of California’s surface water ambient monitoring program. Surface Water Ambient Monitoring Program website. Available: http://www.swrcb.ca.gov/water_issues/programs/swamp/tools.shtml. Accessed 2013 May 23.

[34] USEPA (2012) Recreational water quality criteria. Washington D.C.: US Environmental Protection Agency Office of Water Regulations and Standards. 73 p.

[35] CVRWQCB (2011) The water quality control plan (basin plan) for the California Regional Water Quality Control Board, Central Valley region. Sacramento: Central Valley Regional Water Quality Control Board. 148 p.

[36] LRWQCB (2005) Water quality control plan for the Lahontan region. South Lake Tahoe: Lahontan Regional Water Quality Control Board. 55 p.

[37] NCRWQCB (2011) Water quality control plan for the North Coast region. Santa Rosa: North Coast Regional Water Quality Control Board. 13 p.

[38] Cline C (1973) The effects of forest fertilization on the Tahuya River, Kitsap Peninsula, Washington. Olympia: Washington State Department of Ecology. 55 p.

[39] MacDonald LH, Smart AW, Wissmar RC (1991) Monitoring guidelines to evaluate effects of forestry activities on streams in the Pacific Northwest and Alaska, EPA 910/9–91–001. Seattle: US Environmental Protection Agency. 180 p.

[40] Mackenthun KM (1973) Toward a cleaner aquatic environment. Washington, D.C.: US Environmental Protection Agency. 289 p.

[41] USEPA (1986) Quality criteria for water: 1986. Washington, D.C.: US Environmental Protection Agency Office of Water Regulations and Standards. 477 p.

[42] USEPA (1987) Surface water monitoring: A framework for change. Washington, D.C.: US Environmental Protection Agency Office of Water Regulations and Standards. 41 p.

[43] USEPA (2000) Ambient water quality criteria recommendations: Rivers and streams in Ecoregion II. Washington, D.C.: US Environmental Protection Agency Office of Water Regulations and Standards. 120 p.

[44] Rabe-Hesketh S, Skrondal A (2008) Multilevel and longitudinal modeling using stata. College Station: Stata Press. 562 p.

[45] Pinheiro JC, Bates DM (2000) Mixed-effects models in S and S-PLUS. New York: Springer-Verlag. 528 p.

[46] Cameron AC, Trivedi PK (1998) Regression analysis of count data. Cambridge: Cambridge University Press.

[47] Long JS (1997) Regression models for categorical and limited dependent variables. Thousand Oaks: Sage.

[48] StataCorp (2009) Stata Statistical Software: Release 11. College Station: StataCorp LP.

[49] Vuong QH (1989) Likelihood ratio tests for model selection and non-nested hypotheses. Econometrica 57: 307–333.

[50] Williams RL (2000) A note on robust variance estimation for cluster-correlated data. Biometrics 56: 645–646. 10877330 10.1111/j.0006-341x.2000.00645.x

[51] Campbell JL, Hornbeck JW, McDowell WH, Buso DC, Shanley JB, et al (2000) Dissolved organic nitrogen budgets for upland, forested ecosystems in New England. Biogeochemistry 49: 123–142.

[52] Adams MJ, Pearl CA, McCreary B, Galvan SK, Wessell SJ, et al (2009) Short-term effect of cattle exclosures on Columbia spotted frog (*Rana luteiventris*) populations and habitat in northeastern Oregon. J Herpetol 43: 132–138.

[53] Green MB, Fritsen CH (2006) Spatial variation of nutrient balance in the Truckee River, California-Nevada. J Am Water Resour As 42: 659–674.

[54] Leonard RL, Kaplan LA, Elder JF, Coats RN, Goldman CR (1979) Nutrient transport in surface runoff from a subalpine watershed, Lake Tahoe Basin, California. Ecol Monogr 49: 281–310.

[55] Peterson BJ, Wollheim WM, Mulholland PJ, Webster JR, Meyer JL, et al (2001) Control of nitrogen export from watersheds by headwater streams. Science 292: 86–90. 11292868 10.1126/science.1056874

[56] Hill BH, McCormick FH, Harvey BC, Johnson SL, Warren ML, et al (2010) Microbial enzyme activity, nutrient uptake and nutrient limitation in forested streams. Freshwater Biol 55: 1005–1019.

[57] Ahearn DS, Sheibley RW, Dahlgren RA, Keller KE (2004) Temporal dynamics of stream water chemistry in the last free-flowing river draining the western Sierra Nevada, California. J Hydrol 295: 47–63.

[58] Bernal S, Butturini A, Sabater F (2005) Seasonal variations of dissolved nitrogen and DOC : DON ratios in an intermittent Mediterranean stream. Biogeochemistry 75: 351–372.

[59] Miller WW, Johnson DW, Denton C, Verburg PSJ, Dana GL, et al (2005) Inconspicuous nutrient laden surface runoff from mature forest Sierran watersheds. Water Air Soil Poll 163: 3–17.

[60] van Verseveld WJ, McDonnell JJ, Lajtha K (2008) A mechanistic assessment of nutrient flushing at the catchment scale. J Hydrol 358: 268–287.

[61] Kaushal SS, Lewis WM (2005) Fate and transport of organic nitrogen in minimally disturbed montane streams of Colorado, USA. Biogeochemistry 74: 303–321.

[62] Doran JW, Schepers JS, Swanson NP (1981) Chemical and bacteriological quality of pasture runoff. J Soil Water Conserv 36: 166–171.

[63] Gary HL, Johnson SR, Ponce SL (1983) Cattle grazing impact on surface-water quality in a Colorado Front Range stream. J Soil Water Conserv 38: 124–128.

[64] Schepers JS, Francis DD (1982) Chemical water-quality of runoff from grazing land in Nebraska. 1. Influence of grazing livestock. J Environ Qual 11: 351–354.

[65] Tiedemann AR, Higgins DA, Quigley TM, Sanderson HR, Marx DB (1987) Responses of fecal-coliform in streamwater to 4 grazing strategies. J Range Manage 40: 322–329.

[66] Allan JD (1995) Stream Ecology: Structure and function of running waters. London: Chapman & Hall. 388 p.

[67] Edwards DR, Coyne MS, Daniel TC, Vendrell PF, Murdoch JF, et al (1997) Indicator bacteria concentrations of two northwest Arkansas streams in relation to flow and season. T ASAE 40: 103–109.

[68] Lau YL, Liu D (1993) Effect of flow-rate on biofilm accumulation in open channels. Water Res 27: 355–360.

[69] Krupa M, Tate KW, Van Kessel C, Sarwar N, Linquist B (2011) Water quality in rice-growing watersheds in a Mediterranean climate. Agr Ecosyst Environ 144: 290–301.

[70] Tate KW, Lancaster DL, Lile DF (2007) Assessment of thermal stratification within stream pools as a mechanism to provide refugia for native trout in hot, arid rangelands. Environ Monit Assess 124: 289–300. 16897516 10.1007/s10661-006-9226-5

[71] Alm EW, Burke J, Hagan E (2006) Persistence and potential growth of the fecal indicator bacteria, *Escherichia coli*, in shoreline sand at Lake Huron. J Great Lakes Res 32: 401–405.

[72] Byappanahalli MN, Whitman RL, Shively DA, Ting WTE, Tseng CC, et al (2006) Seasonal persistence and population characteristics of *Escherichia coli* and enterococci in deep backshore sand of two freshwater beaches. J Water Health 4: 313–320. 17036839 10.2166/wh.2006.518

[73] Roszak DB, Colwell RR (1987) Survival strategies of bacteria in the natural-environment. Microbiol Rev 51: 365–379. 3312987 10.1128/mr.51.3.365-379.1987 PMC373117

[74] Clark ML, Norris JR (2000) Occurrence of fecal coliform bacteria in selected streams in Wyoming,1990–99: Water resources investigations report 00–4198. Cheyenne: US Geological Survey. 8 p.

[75] Schnabel WE, Wilson T, Edwards R, Stahnke G, Maselko M, et al (2010) Variability, seasonality, and persistence of fecal coliform bacteria in a cold-region, urban stream. J Cold Reg Eng 24: 54–75.

[76] Roche LM, Latimer AM, Eastburn DJ, Tate KW (2012) Cattle grazing and conservation of a meadow-dependent amphibian species in the Sierra Nevada. PloS One 7: 1–11. 10.1371/journal.pone.0035734 PMC3338456 22558211

[77] Knox AK, Tate KW, Dahgren RA, Atwill ER (2007) Management reduces *E. coli* in irrigated pasture runoff. Calif Agr 61: 159–165.

[78] Jamieson R, Joy DM, Lee H, Kostaschuk R, Gordon R (2005) Transport and deposition of sediment-associated *Escherichia coli* in natural streams. Water Res 39: 2665–2675. 15979685 10.1016/j.watres.2005.04.040

[79] Jamieson RC, Gordon RJ, Tattrie SC, Stratton GW (2003) Sources and persistence of fecal coliform bacteria in a rural watershed. Water Qual Res J Can 38: 33–47.

[80] Sherer BM, Miner JR, Moore JA, Buckhouse JC (1992) Indicator bacterial survival in stream sediments. J Environ Qual 21: 591–595.

[81] Stephenson GR, Rychert RC (1982) Bottom sediment - a reservoir of *Escherichia-coli* in rangeland streams. J Range Manage 35: 119–123.

[82] Van Donsel DJ, Geldreich EE (1971) Relationships of *Salmonellae* to fecal coliforms in bottom sediments. Water Res 5: 1079–1087.

[83] Porath ML, Momont PA, DelCurto T, Rimbey NR, Tanaka JA, et al (2002) Offstream water and trace mineral salt as management strategies for improved cattle distribution. J Anim Sci 80: 346–356. 11881924 10.2527/2002.802346x

[84] Lewis DJ, Atwill ER, Lennox MS, Pereira MDG, Miller WA, et al (2009) Reducing microbial contamination in storm runoff from high use areas on California coastal dairies. Water Sci Technol 60: 1731–1743. 19809136 10.2166/wst.2009.561

