# Supplementary material for: Water Quality Conditions Associated with Cattle Grazing and Recreation on National Forest Lands
Source: PLoS One. 2013 Jun 27;8(6):e68127. doi: 10.1371/journal.pone.0068127 (PMC3694922; doi:10.1371/journal.pone.0068127)
Supplement: Table S1 — Geographic characteristics, study year precipitation, cattle grazing management, and water quality sample collection sites and sample numbers for 12 U.S. Forest Service grazing allotments in northern California enrolled in this cross-sectional longitudinal study of stream water quality between June and November 2011. (DOCX) [file pone.0068127.s001.docx]

| Allotment | Area | Elevation | Dominant | Precipitation^b^ | AUM^c^ | Cow-Calf | Cattle | Cattle | Grazing | Sample | Sample |
| --- | --- | --- | --- | --- | --- | --- | --- | --- | --- | --- | --- |
|  | (km²) | (m) | Soil^a^ | cm (%) |  | Pairs^d^ | Turn On^e^ | Turn Off^f^ | Standard^g^ | Sites | Number |
| 1 | 29 | 1353-2304 | Xerepts | 147 (104) | 317 | 80 | 8/2/11 | 11/9/11 | 60 | 17 | 88 |
| 2 | 131 | 1000-2374 | Xerepts | 146 (125) | 311 | 108 | 8/6/11 | 10/17/11 | 60 | 8 | 44 |
| 3 | 139 | 1646-2751 | Xeralfs | 149 (105) | 84 | 31 | 8/16/11 | 10/23/11 | 60 | 12 | 57 |
| 4 | 162 | 1128-1707 | Xerands | 167 (116) | 1102 | 207 | 6/21/11 | 10/31/11 | 60 | 17 | 83 |
| 5 | 85 | 207-1387 | Xerepts | 159 (88) | 301 | 55 | 6/16/11 | 10/31/11 | 60 | 13 | 44 |
| 6 | 258 | 219-670 | Xerepts | 202 (116) | 669 | 113 | 6/5/11 | 10/31/11 | 60 | 15 | 82 |
| 7 | 49 | 1768-2341 | Xerepts | 160 (145) | 351 | 101 | 7/21/11 | 10/16/11 | 40 | 7 | 40 |
| 8 | 50 | 1760-2345 | Xerolls | 111 (127) | 560 | 280 | 7/14/11 | 9/2/11 | 40 | 15 | 78 |
| 9 | 109 | 1622-2670 | Xerolls | 120 (133) | 738 | 143 | 6/1/11 | 10/4/11 | 40 | 10 | 49 |
| 10 | 31 | 1878-2609 | Xeralfs | 160 (173) | 382 | 105 | 7/22/11 | 10/21/11 | 40 | 18 | 82 |
| 11 | 53 | 1935-2789 | Xerepts | 194 (147) | 234 | 80 | 7/19/11 | 9/30/11 | 40 | 12 | 45 |
| 12 | 207 | 610-2116 | Xeralfs | 168 (151) | 2077 | 440 | 7/1/11 | 10/27/11 | 40 | 11 | 51 |

^a^ Dominant soil suborder identified with soil survey data [16].

^b^ Precipitation realized during the October 1, 2010 through September 30, 2011 water year. Values in parentheses are the percent of 30-year mean annual precipitation realized during the 2010-11 water year [42].

^c^ Animal Unit Month. The dry weight mass of forage required to feed a 1000 lb. cow for a 30 day period. It is the standard unit by which grazing pressure is permitted on U.S. Forest Service grazing allotments.

^d^ Total cow-calf pairs per allotment.

^e^ Date that cattle were released onto the grazing allotment.

^f^ Date that cattle were removed from the grazing allotment.

^g^ Maximum permissible removal of annual herbaceous vegetation production in meadows and riparian areas on the grazing allotment.
