# Supplementary material for: Water Quality Conditions Associated with Cattle Grazing and Recreation on National Forest Lands
Source: PLoS One. 2013 Jun 27;8(6):e68127. doi: 10.1371/journal.pone.0068127 (PMC3694922; doi:10.1371/journal.pone.0068127)
Supplement: Table S2 — Mean, median, and maximum fecal coliform (FC) and E. coli concentrations for 743 stream water samples collected across 155 sample sites on 12 U.S. Forest Service grazing allotments in northern California. All concentrations are reported as colony forming units per 100 ml of sample water (cfu 100 ml−1). (DOCX) [file pone.0068127.s002.docx]

| Allotment | Mean^a^ | | Median | | Maximum | |
| --- | --- | --- | --- | --- | --- | --- |
|  | FC | *E. coli* | FC | *E. coli* | FC | *E. coli* |
|  | (cfu 100 ml^-1^) | (cfu 100 ml^-1^) | (cfu 100 ml^-1^) | (cfu 100 ml^-1^) | (cfu 100 ml^-1^) | (cfu 100 ml^-1^) |
| 1 | 58 ± 10 | 17 ± 5 | 21 | 4 | 504 | 282 |
| 2 | 64 ± 20 | 39 ± 12 | 14 | 6 | 540 | 330 |
| 3 | 30 ± 10 | 17 ± 6 | 11 | 6 | 568 | 312 |
| 4 | 31 ± 7 | 18 ± 5 | 12 | 6 | 530 | 360 |
| 5 | 102 ± 27 | 69 ± 19 | 12 | 3 | 650 | 500 |
| 6 | 46 ± 15 | 21 ± 7 | 8 | 3 | 1134 | 502 |
| 7 | 255 ± 83 | 151 ± 57 | 67 | 33 | 2220 | 1920 |
| 8 | 112 ± 19 | 38 ± 6 | 43 | 26 | 860 | 216 |
| 9 | 114 ± 24 | 63 ± 13 | 36 | 17 | 590 | 340 |
| 10 | 47 ± 6 | 21 ± 4 | 21 | 10 | 260 | 270 |
| 11 | 57 ± 12 | 18 ± 3 | 14 | 6 | 248 | 74 |
| 12 | 190 ± 72 | 94 ± 29 | 48 | 30 | 3460 | 1090 |
| Overall | 82 ± 8 | 40 ± 4 | 21 | 8 | 3460 | 1920 |

^a^ The ‘±’ indicates 1 standard error of the mean.
